# Impact of race on dose selection of molecular-targeted agents in early-phase oncology trials

**DOI:** 10.1038/s41416-018-0102-1

**Published:** 2018-05-24

**Authors:** Tomoya Yokota, Johanna Bendell, Patricia LoRusso, Takahiro Tsushima, Ved Desai, Hirotsugu Kenmotsu, Junichiro Watanabe, Akira Ono, Bhavani Murugesan, Joseph Silva, Tateaki Naito, Jonathan Greenberg, Prasanna Kumar, Yibin Wang, Takahiro Jikoh, Ryota Shiga, David M. Hyman, Alan Loh Ho, David R. Spriggs, Gary K. Schwartz, Mrinal M. Gounder

**Affiliations:** 1 Division of Gastrointestinal Oncology, Shizuoka Cancer Center, 1007 Shimonagakubo, Nagaizumi-cho, Sunto-gun, Shizuoka 411-8777 Japan; 2 Sarah Cannon Research Institute/Tennessee Oncology, 250 25th Ave N Suite 200, Nashville, TN 37203 USA; 3 Yale University, 55 Park Street, Ste First Floor, New Haven, CT 06519 USA; 4 Memorial Sloan-Kettering Cancer Center and Weill Cornell Medical College, New York, NY 10065 USA; 5 Division of Thoracic Oncology, Shizuoka Cancer Center, 1007 Shimonagakubo, Nagaizumi-cho, Sunto-gun, Shizuoka 411-8777 Japan; 6 Division of Breast Oncology, Shizuoka Cancer Center, 1007 Shimonagakubo, Nagaizumi-cho, Sunto-gun, Shizuoka 411-8777 Japan; 7 Daiichi Sankyo, Inc., 399 Thornall Street, Edison, NJ 08837 USA; 8 Daiichi Sankyo, Inc., 1-2-58, Hiromachi, Shinagawa-ku, Tokyo, 140-8710 Japan; 9 Division of Hematology/Oncology, Columbia University, 161 Fort Washington Avenue (Floor 9), New York, NY 10032 USA

**Keywords:** Drug development, Targeted therapies

## Abstract

**Background:**

We examined the impact of race on the maximum tolerated doses (MTD) and final approved doses (FAD) of single-agent molecular-targeted agents (MTA) in North America/Europe (NA/EU) and Asia.

**Methods:**

We searched PubMed and regulatory databases to identify targeted drugs approved globally and compared their FAD and MTD in corresponding phase I/II studies conducted separately in NA/EU and Asia. To evaluate this further, we conducted parallel, prospective, first-in-human studies of DS-7423, a dual PI3K/mTOR inhibitor, in patients with advanced solid tumours in the US and Japan. We pooled and compared the pharmacokinetics (PK), pharmacodynamics (PD), toxicity, and efficacy between these populations.

**Results:**

17 MTA were approved in NA/EU and Asia from 2001 to 2015. Recommended phase 2 doses (RP2D) were identical across races in 14 of 17 (80%) studies and differences were not clinically meaningful. FAD were identical across all regions. 42 and 27 patients from US and Japan, respectively, were enrolled in the phase I studies of DS-7423. Despite differences in race, body weight, and body mass index, the RP2D were 240 mg/day with no differences in toxicities, PK, PD, or efficacy.

**Conclusions:**

Conducting separate clinical trials of single-agent MTA in Caucasian and Asian populations may be redundant.

## Introduction

The advent of biomarker-driven or precision medicine trials has been transformative for the oncology drug development and approval process. Increasingly, the traditional model for drug development (phases I, II, and III) is being abandoned for a “seamless” approach where first-in-human trials investigate dose and activity in a variety of cancers (“basket” studies), which then become the basis for drug approval.^1^ Drugs that have highlighted this process are ALK inhibitors in ALK/ROS1 rearranged non-small cell lung cancer (NSCLC), EGFR inhibitors in EGFR T790M NSCLC, and pembrolizumab in tumours with mismatch repair deficiency (dMMR) or microsatellite instability-high, among others.^2–5^ A major challenge with biomarker selective trials is the identification of appropriate patients, particularly when the biomarker is rare and/or requires technically sophisticated methods to detect. This has been underscored in recent clinical trials of NTRK inhibitors in advanced cancers harboring TRK fusion, a genetic event that is exceedingly rare (<1%).^6^ Such trials require a priori knowledge of genetic aberrations and thus are only feasible in developed countries with large populations and extensive resources. As all cancers are increasingly redefined by genetic signatures and less by the cell of origin, even common cancers are being categorised as rare entities. The emerging paradigm of a strategy involving a seamless transition from phase I to pivotal drug development is only logistically and economically sustainable when there is increased global participation along with seamless extrapolation of results from one region/population to another.

Under existing regulatory guidelines, specifically the International Conference on Harmonization; Ethnic Factors in the Acceptability of Foreign Clinical Data (ICH R5), a drug development strategy should adequately address intrinsic (e.g., pharmacogenomics) and extrinsic (e.g., diet) factors that are potentially unique to a race and/or ethnic population and which may influence drug metabolism, safety, and efficacy.^7^ The ICH R5 guidelines, which are jointly issued by the Food and Drug Administration (FDA, USA), European Medicines Agency (EMA, EU), and Pharmaceuticals and Medical Devices Agency (PMDA, Japan), are intended to allow meaningful and timely extrapolation of late-phase trial results from one population to another in order to eliminate redundancies and expedite rapid drug access to patients. However, this is typically contingent on the early-phase clinical trials being repeated in Asia to discern the effect of race on pharmacokinetics (PK). While ethnic factors have led to different drug labels and indications in a wide range of non-oncological diseases, we sought to evaluate the impact of race on PK, pharmacodynamics (PD), safety, and dose selection of molecular-targeted agents in oncology.^8^

## Patients and methods

### Study design

We conducted a search of regulatory databases (FDA, EMA, and PMDA) and PubMed to identify all molecular-targeted agents approved as monotherapy in solid tumours in all three regions: North America/Europe (NA/EU) and Asia. Molecular agents are defined as drugs that target extracellular or intracellular mechanisms different from conventional chemotherapy, such as DNA or cell division machinery. We excluded new indications for previously approved drugs; combination studies; and cytotoxic, hormone, antibody, vaccine, and immunotherapy drugs. For each approved drug, we noted the final approved dose (FAD), approval date, and the corresponding maximum tolerated dose (MTD) and recommended phase 2 dose (RP2D) from the phase Ib studies conducted independently in NA/EU and Asia.

We conducted two independent, prospective, first-in-human phase I studies of DS-7423, a dual PI3K/mTOR inhibitor, in patients with advanced, refractory solid tumours in the US (U101 study) and Japan (J102 study); age ≥18 years in the US and ≥20 years in Japan; and Eastern Cooperative Oncology Group performance status (ECOG PS) 0–1. Key exclusion criteria included fasting glucose >126 mg/dL (>7 mmol/L); HbA1c >7.0% for the US and HbA1c >6.5% for Japan; history of diabetes mellitus (type I or II); concomitant use of chronic systemic corticosteroids, or prior toxicities from a dual PI3K/mTOR inhibitor (Supplementary Table 1). Studies were approved by the Institutional Review Board at the participating institutions and regulatory authorities. Written informed consent in English and Japanese was obtained from all patients prior to enrollment. The study followed the Declaration of Helsinki and good clinical practice guidelines and was registered with ClinicalTrials.gov (NCT01364844) and with ClinicalTrials.jp (Japic CTI, 12766).

### Endpoints

The primary objective was to assess the safety and tolerability to determine the MTD and RP2D of DS-7423 in US and Japanese patients. Secondary and exploratory objectives were to evaluate PK, tumour response, and PD effects. Treatment-emergent adverse events (TEAE) were graded using the Common Terminology Criteria for Adverse Events version 4.0. A dose-limiting toxicity (DLT) was defined as a grade ≥3 clinically significant toxicity, with the exceptions of grade ≥4 anaemia and prolonged grade 3 neutropenia. Both studies were open-label, phase I dose-escalation studies. In Japan, the study was conducted at a single centre, while the US study was conducted at three sites. In Japan, dose escalation of DS-7423 was guided by the modified continuous reassessment method (mCRM) using a Bayesian logistic regression model following the escalation with overdose control (EWOC) principle at a starting dose of 4 mg once daily.^9^ In the US, an accelerated titration design was used at low doses followed by a mCRM with EWOC. Briefly, the dose was escalated based on safety data in a single patient until a grade 2 or higher toxicity was observed during cycle 1 or the DLT period (days 1–28). In both studies, DS-7423 was administered orally once daily until patients experienced unacceptable treatment-related toxicity or disease progression. Prophylactic granulocyte colony-stimulating factor and antiemetics were not permitted during cycle 1.

### PK, PD, toxicity, and tumour assessments

In both studies, the PK profile of DS-7423 was characterised during cycle 1 on days 1 and 8. For PD analysis, glucose, C-peptide, and Akt phosphorylation were measured in platelet-rich plasma collected at predose, 1, 2, 4, 6, and 24 h after dosing on days 1 and 15. Tumour assessment was performed every two cycles using Response Evaluation Criteria in Solid Tumours (RECIST) version 1.1 in both studies. 18F-Fluoro-2-deoxy-D-glucose positron emission tomography ([18F]-FDG) was performed as an exploratory endpoint at baseline and on day 4.^10^

### Statistical analysis

The data cut-off for the primary analysis occurred after all patients had either discontinued the study or completed 6 months of study drug treatment. Descriptive statistics were provided for selected demographic, safety, PK, and PD data by dose and time as appropriate. Descriptive statistics on continuous data included means, medians, standard deviations, and ranges, while categorical data were summarised using frequency counts and percentages. No inferential analysis to compare dose levels was performed due to limited sample sizes. The WinNonlin software was used for PK analysis.

## Results

### Globally approved MTA

From 2001 to 2015, 17 MTA were approved by PMDA (Japan) and FDA (US) or EMA (EU) as monotherapy for advanced solid tumours (Table 1).^11–44^ All MTA were approved at identical doses in North America, Europe, and Asia (17/17, 100%). The corresponding phase 1/b studies for each of these drugs were conducted separately in NA/EU and Asia for 16 of 17 (94%) drugs. The exception was osimertinib, for which no phase I study was conducted in Asia, but PMDA approval was granted based on a global phase III trial with Japanese participation. Sixteen first-in-human studies were initially performed in NA/EU, and these were replicated to confirm safety and MTD in Asian patients. Key PK parameters of some approved molecular-targeted drugs in Caucasian and Asian at the MTD were shown in Supplementary Table 2; however, due to lack of availability of raw data statistical calculations were not performed. Overall, the RP2D was identical in 13 of 16 (81%) studies conducted in NA/EU and Asia. In the other three studies, the drugs involved were temsirolimus, everolimus, and lapatinib, but the discordance was not related to race. The lag time for approval between NA/EU and Asia was a median of 2 years (−0.8 to 4.5 years). Gefitinib received approval in Asia 8 months prior to in NA/EU and vandetanib received it 4.5 years later in Asia.

**Table 1:** Maximum tolerated doses of approved drugs in Japan, US, and EU

Name of drug (references)	RP2D (Japan)	RP2D (Western)	Concordance (Phase I)	PMDA approved dose and date	FDA or EMA approved dose and date	Concordance^a^ (approved)	Delay in approval in Japan (years)
Gefitinib	250 and 500 mg	250 and 500 mg	Concordant	250 mg QD 7/5/2002	250 mg QD 5/5/2003	Concordant	−0.8
Erlotinib	150 mg QD	150 mg QD	Concordant	150 mg QD 10/19/2007	150 mg QD 11/18/2004	Concordant	2.9
Sorafenib	400 mg BID	400 mg BID	Concordant	400 mg BID 1/25/2008	400 mg BID 12/20/2005	Concordant	2
Sunitinib	50 mg QD	50 mg QD	Concordant	50 mg QD 4/16/2008	50 mg QD 1/26/2006	Concordant	2.1
Crizotonib	250 mg BID	250 mg BID	Concordant	250 mg BID 3/20/2012	250 mg BID 8/26/2011	Concordant	0.5
Axitinib	5 mg BID	5 mg BID	Concordant	5 mg BID 6/29/2012	5 mg BID 1/27/2012	Concordant	0.4
Pazopanib	800 mg or 1000 mg QD	800 mg QD	Concordant	800 mg QD 9/28/2012	800 mg QD 10/19/2009	Concordant	2.9
Regorafenib	160 mg QD	160 mg QD	Concordant	160 mg QD 3/25/2013	160 mg QD 9/27/2012	Concordant	0.5
Afatinib	50 mg QD	50 mg QD	Concordant	40 mg QD 1/17/2014	40 mg QD 7/12/2013	Concordant	0.5
Vemurafenib	960 mg BID	960 mg BID	Concordant	960 mg BID 12/26/2014	960 mg BID 8/17/2011	Concordant	2.3
Lenvatinib	24 mg QD	25 mg QD	Concordant	24 mg QD 3/26/2015	24 mg QD 2/13/2015	Concordant	0.1
Vandetanib	300 mg QD	300 mg QD	Concordant	300 mg QD 9/28/2015	300 mg QD 4/6/2011	Concordant	4.5
Ceritinib	750 mg QD	750 mg QD	Concordant	750 mg QD 3/28/2016	750 mg QD 4/29/2014	Concordant	1.9
Lapatinib	1500 mg QD	1250 mg QD	Different	1250 mg QD 4/22/2009	1250 mg QD 3/13/2007	Concordant	2
Everolimus	10 mg QD	5 or 10 mg QD or 20–50 mg weekly	Different	10 mg QD 1/20/2010	10 mg QD 3/30/2009	Concordant	0.8
Temsirolimus	15 mg/m^2^ weekly	Weekly doses of 25, 75, and 250 mg	Different	25 mg weekly 7/23/2010	25 mg weekly 5/30/2007	Concordant	3.1
Osimertinib	Not done	80 mg QD	NA	80 mg QD 3/28/2016	80 mg QD 11/13/2015	Concordant	0.3

*RP2D* recommended phase 2 dose.

^a^In early-phase and late-phase studies, drugs that had identical doses or where the maximum tolerated dose was not reached in both regions were designated as “concordant”, while those for studies where the final doses differed were “discordant”

### Patient characteristics in DS-7423 studies

In total, 42 and 27 patients were enrolled from June 2011 to June 2013 and from April 2012 to June 2014 in the US and Japan, respectively (Table 2). There were no differences in median age, sex, ECOG performance status, or number of prior antineoplastic regimens between the US and Japanese studies. Regarding race, all patients identified themselves as Asian in the Japanese study, whereas 91% identified themselves as Caucasian in the US study. Body weight (BW), body mass index (BMI), and body surface area (BSA) were higher in the US study. There were no differences in baseline laboratory characteristics between the two studies (data not shown).

**Table 2:** Patient characteristics in the DS-7423 study

		US cohort		Japanese cohort		Total	
		(*n* = 42)		(*n* = 27)		(*n* = 69)	
*Characteristics*		*n*	%	*n*	%	*n*	%
Age (years) [mean (range)]		60.4 (18–87)		61.5 (40–73)			
Sex	Male	19	45.2	14	51.9	33	47.8
	Female	23	54.8	13	48.1	36	52.2
Performance status (ECOG)	0	15	35.7	9	33.3	24	34.8
	1	27	64.3	18	66.7	45	65.2
Prior antineoplastic regimens		42	100	25	92.6	67	97.1
No. of prior regimens	Median	4		3			
	Range	1–15		0–9			
Patients with ≥3 prior regimens		33	78.6	15	55.6	48	69.6
Tumour type	Colorectal	10	23.8	3	11.1	13	18.8
	Breast	3	7.1	3	11.1	6	8.7
	Oesophageal	0	0	6	22.2	6	8.7
	Non-small cell lung	4	9.5	1	3.7	5	7.3
	Thymic cancer or thymoma	0	0	4	14.8	4	5.8
	Cholangiocarcinoma	3	7.1	1	3.7	4	5.8
	Ovarian	2	4.8	2	7.4	4	5.8
	Pancreatic	3	7.1	0	0	3	4.4
	Squamous cell carcinoma	3	7.1	0	0	3	4.4
	Mesothelioma	1	2.4	2	7.4	3	4.4
	Carcinoid	0	0	2	7.4	2	2.9
	Prostate	2	4.8	0	0	2	2.9
	Gastric	0	0	2	7.4	2	2.9
	Others^a^	11	26.2	1	3.7	12	17.4
Race	White	38	90.5	0	0	38	55.1
	African-American	2	4.8	0	0	2	2.9
	Asian	2	4.8	0	0	2	2.9
	Japanese	0	0	27	100	27	39.1
Body weight (kg) [mean ± SD (range)]		79.23 ± 23.88 (38.2–141.4)		56.7 ± 10.84 (33.3–72)			
BMI (kg/m^2^) [mean ± SD (range)]		27.68 ± 6.875 (14.0–46.1)		21.6 ± 3.4 (14.7–27.7)			
BSA^b^ ± SD		1.88 ± 0.292		1.59 ± 0.181			
Creatinine clearance^c^ (ml/min) [mean ± SD]		94.88 ± 52.954		86.7 ± 27.31			

*ECOG* Eastern Cooperative Oncology Group, *SCC* squamous cell carcinoma, *BSA* body surface area, *BMI* body mass index, *SD* standard deviation.

^a^ One patient each with acrospiroma (not a cancer), adenoid cystic carcinoma, bladder, endometrial, large cell carcinoma of lung, laryngeal, melanoma, renal, sarcoma, small bowel carcinoma, thyroid carcinoma, and peritoneal carcinoma.

^b^ BSA was calculated using the formula of Du Bois

^c^ Cockcroft and Gault’s formula

### Toxicities in DS-7423 studies

In the US study, patients received DS-7423 at a dose level of 2, 4, 8, 16, 28, 48, 80, 160, 240, or 320 mg. One patient was enrolled at each dose level from 2 to 8 mg, as no grade ≥2 non-disease-related toxicity or DLT was observed during cycle 1. The accelerated titration design was then shifted to mCRM with EWOC dose escalation for the cohorts with a dose of 16 mg or above, owing to grade 2 blood alkaline phosphatase. DLT occurred in 4 of 34 DLT-evaluable patients (Supplementary Table 3): grade 3 DLT included rash (48 mg), stomatitis (240 mg), and fatigue and dehydration (240 mg). The 240-mg dose level was defined as the MTD in the US. Similarly, in the Japanese study, patients received DS-7423 at dose levels of 4, 8, 16, 32, 56, 96, 160, and 240 mg. Grade 3 DLT included lung infections and hyperglycaemia in the 240-mg cohort. The MTD was determined to be 240 mg in Asian patients (Supplementary Table 3).

Table 3 summarises the TEAE, of which the total incidence was ≥10% of patients in both studies combined, or clinically significant or severe (≥grade 3) TEAE. Among all cohorts, the most common all-grade TEAE related to study treatment were diarrhoea (56.5%), fatigue (55.1%), decreased appetite (49.3%), rash (33.3%), and stomatitis (33.3%). Although nausea, vomiting, and dysgeusia were also reported, all events were grade 1 or 2. DS-7423-related ≥grade 3 TEAE were rare. Overall, there were no remarkable differences in the toxicities between the Caucasian and Asian patients.

**Table 3:** Treatment-emergent adverse events

Adverse event	4–32 mg Japanese cohort (*n* = 12)	2–28 mg US cohort (*n* = 10)	56–96 mg Japanese cohort (*n* = 6)	48–80 mg US cohort (*n* = 11)	160 mg Japanese cohort (*n* = 3)	160 mg US cohort (*n* = 4)	240 mg Japanese cohort (*n* = 6)	240 mg US cohort (*n* = 9)	320 mg US cohort (*n* = 8)	All (*n* = 69)
Diarrhoea	6	5	4 (96 mg 1 G3)	4	2 (1 G3)	4	5	5	4	39 (56.5%)
Nausea	6	6	1	6	3	2	6	4	5	39 (56.5%)
Fatigue	5	7 (2, ≥G3)	3	5 (1, ≥G3)	1	4	2	5 (1, ≥G3)	6 (1, G3)	38 (55.1%)
Decreased appetite	7	3	2	2	3 (1 G3)	3	4	5 (1, ≥G3)	5	34 (49.3%)
Vomiting	4	4	1	3	2	2	3	3	4	26 (37.7%)
Rash	3	2	4	4 (1, G3)	2	0	5	0	3	23 (33.3%)
Stomatitis	3	1	1	3	2	1	6	5 (1, G3)	1	23 (33.3%)
Dysgeusia	3	0	1	2	2	1	3	2	5	19 (27.5%)
Constipation	3	3	1	0	1	2	1	1	1	13 (18.8%)
Dry mouth	0	3	1	3	0	0	1	3	2	13 (18.8%)
Edema	2	2	1	1	1	0	3	1	1	12 (17.4%)
Dyspnea	2	2	0	1	1	0	0	3	2	12 (17.4%)
Hyperglycaemia	0	0	2 (56 mg 1 G3, 96 mg 1 G3)	2	1 (1 G3)	1	1 (1 G4)	2	3	12 (17.4%)
Cough	1	2	1	3	2	0	1	0	1	11 (15.9%)
Back pain	1	5	0	1	0	0	1	3	0	11 (15.9%)
Pyrexia	0	1	0	2	1	0	2	2	2	10 (14.5%)
Abdominal pain	1	0	1	1	1	1	1	4	0	10 (14.5%)
Blood creatinine increased	1	0	0	0	0	2	0	2	2	7 (10.1%)
AST increased	0	3 (1, ≥G3)	1	1	0	0	0	2, ≥G3	0	7 (10.1%)
Dehydration	0	0	0	1, G3	0	1	0	1	3 (1, G3)	6 (8.7%)
Lung infection/ pneumonia	0	1, G3	0	0	0	0	1 (1 G3)	1	1, G3	4 (5.8%)

Treatment-emergent AE (TEAE) shown here met either of the following criteria: (i) total incidence of ≥10% of the total number of subjects in both studies combined, and (ii) clinically significant or severe (≥G3) TEAE

A DLT regarding liver function was defined as follows: (i) grade 4 alanine aminotransferase (ALT)/aspartate aminotransferase (AST) elevation; (ii) >5× upper limit of normal (ULN) ALT/AST elevation for more than 3 days in cases without liver metastasis; (iii) >5× ULN ALT/AST elevation with grade ≥2 hyperbilirubinemia; (iv) >5× ULN ALT/AST elevation for more than 3 days in cases with liver metastasis and whose baseline ALT/AST is ≤3× ULN; and (v) >8× ULN ALT/AST elevation for more than 3 days in cases with liver metastasis and whose baseline ALT/AST is >3× ULN and ≤5× ULN

### PK in DS-7423 studies

**Fig. 1:**
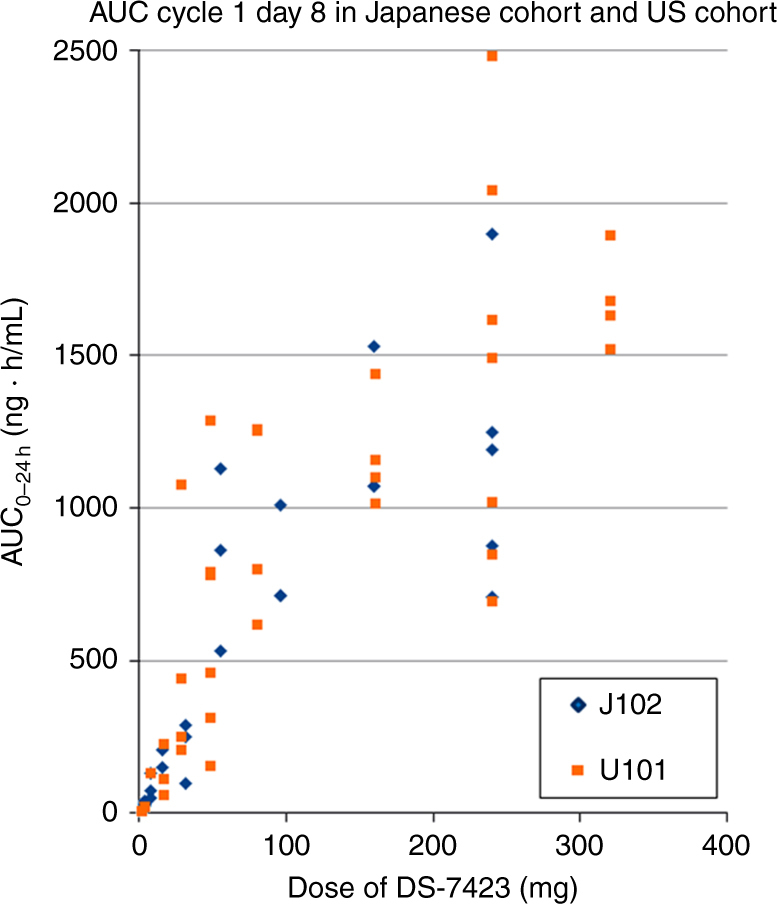
AUC plots 8 h after dosing on day 8, cycle 1, in US cohort and Japanese cohort

Mean concentration, maximum concentration (*C*_max_), and area under the curve (AUC) of DS-7423 increased in dose-dependent manners over the range from 16 to 320 mg in both Caucasian and Asian populations (Supplementary Fig. 1). The predose concentrations on days 15 and 21 of cycle 1 and on day 1 of cycle 2 suggested no remarkable drug accumulation. Importantly, at the MTD of 240 mg, there were no significant differences in AUC and *C*_max_ between the two populations (Fig. 1). Across dose groups, the median time to peak concentration (*T*_max_) was approximately 2 to 4 h. The median half-life of DS-7423 was 12 h at the 240-mg dose (MTD). Taking these findings together, DS-7423 exhibited similar PK parameters between Asian and Caucasian patients (Table 4).

**Table 4:** Comparison of the pharmacokinetics (AUC, *C*_max_, half-life, and *T*_max_) of DS-7423 between US cohort and Japanese cohort

Dose level	Study	Mean AUCtau [ng h/mL]	Mean *C*_max_ [ng/mL]	*T*_1/2_ [h]	*T*_max_ [h]
4	US cohort (*n* = 1)	21.86	4	6–7	2–4
	Japanese cohort (*n* = 3)	31.1	3	6–7	2–4
8	US cohort (*n* = 1)	131	20	6–7	2–4
	Japanese cohort (*n* = 3)	84.2	10	6–7	2–4
16	US cohort (*n* = 3)	133.5	20	6–7	2–4
	Japanese cohort (*n* = 2)	178	18	6–7	2–4
160	US cohort (*n* = 4)	1180.5	59	6–7	2–4
	Japanese cohort (*n* = 3)	1223.3	61	6–7	2–4
240	US cohort (*n* = 7)	1458.2	92.0	12	2–4
	Japanese cohort (*n* = 5)	1184.8	83.6	12	2–4

*AUC* area under the curve

### PD in DS-7423 studies

**Fig. 2:**
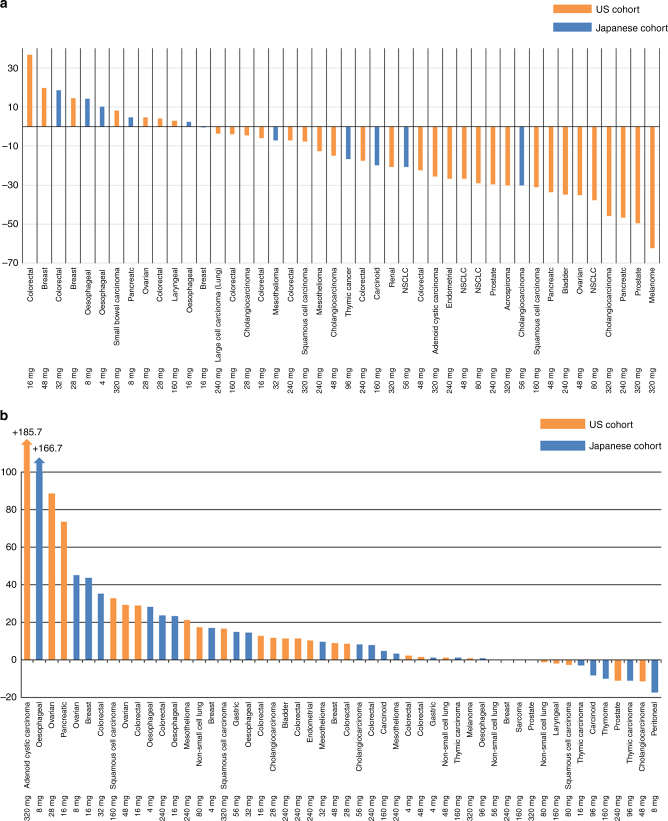
**a** Waterfall plots of the percent change in tumour [18F]-FDG standardised uptake value (SUV) on day 4 from baseline (*n* = 44). **b** Waterfall plots of the percent change in the sum of the longest diameter of the target lesions from baseline to the first CT examination (*n* = 53)

Hyperglycaemia, a known on-target effect of PI3K-Akt-mTOR inhibitors, was observed as early as 4 h after dosing and decreased toward the baseline level at 24 h. This was observed indirectly by a decrease in [18F]-FDG-PET uptake as early as day 4.^45^ Totals of 16 of 33 patients (48.5%) and 4 of 11 (36.4%) patients had a partial metabolic response in Caucasian and Asian patients, respectively (Fig. 2a). In both studies, there was a trend towards greater inhibition with higher doses (Supplementary Fig. 2A, B). Akt phosphorylation in plasma was evaluated at pre-treatment and post-treatment time points in 32 patients. Akt phosphorylation was inhibited compared with that at baseline, although there was variability in the change from baseline (Supplementary Fig. 3).

### Efficacy in DS-7423 studies

A waterfall plot revealed that there was no PR or CR by RECIST 1.1 in either study (Fig. 2b). Overall, 5 of 27 patients in the US and 5 of 25 patients in Japan had tumour shrinkage and durable disease control in peritoneal (8 mg), thymic (16 and 96 mg), laryngeal (160 mg), and prostate (240 mg) cancers, cholangiocarcinoma (48 mg), non-small cell lung and squamous cell carcinomas (80 mg), carcinoid (96 mg), thymoma (160 mg), and sarcoma (160 mg) (Supplementary Fig. 4A, B).

## Discussion

It is estimated that >8 million cancer-related deaths occur annually, and Asia is estimated to account for half the global burden of cancer.^46^ There is an urgent need for a global drug development and approval strategy to decrease cancer-related morbidity and mortality worldwide. Although the ICH E5 guidance (1998) has been in place to meaningfully and rapidly extrapolate clinical data from one race/ethnicity to another, our study found a median lag of 2 years for drug approval between North America/Europe and Asia for single-agent MTA. Moreover, in a 15-year period (2001–2015), every approved dose of an MTA was identical between Caucasian and Asian patients. When we evaluated the corresponding phase Ib studies of each of the drugs conducted independently in NA/EU and Asia, there were no differences in the MTD/RP2D between the two populations. The “discrepancies” (3/16, 19%) between the NA/EU and Japan were noted for temsirolimus, everolimus, and lapatinib. However, these differences were not due to the contribution of race to drug pharmacology. Instead, for all three first-in-human studies conducted in NA/EU, the final MTD and RP2D were inconclusive and problematic.^11,33,35^ For example, in phase 1 study of temsirolimus in US and European countries, the dose escalation over 220 mg/m^2^ dose-level was stopped because of grade 3 depression, grade 3 asthenia and grade 3 stomatitis, although the formal definition of MTD was not met. However, inhibition of the target mTOR kinase and subsequent clinical benefit were achieved below dose levels that result in DLT.^35^ Therefore, weekly doses of 25, 75, and 250 mg were tested in phase II trials in patients with breast and renal cancer. Thus, RP2D in western countries were not based on formal definitions of MTD. Based on above preceding phase 2 and phase 3 trial, the starting dose in Japanese phase 1 trial was set to be 15 mg/m^2^, correspond to flat doses of 25 mg.^16^ In case of everolimus, continuous daily dosing (5 or 10 mg QD) has been found to result in a more profound and sustained inhibition of mTOR than that achieved with an intermittent weekly schedule (20–50 mg weekly).^47,48^ Therefore, daily doses alone up to 10 mg was assessed in Japanese phase 1 study.^34^ Thus, efficacy was observed over a wide dose range, and there was no relationship between drug exposures and efficacy. This suggested that the biologically effective dose was lower than the MTD and therefore multiple RP2D doses were explored. Since these studies were repeated in Asia with a priori knowledge, the investigators recommended a lower RP2D based on pharmacodynamic endpoints. Such a clinically insignificant difference is further reflected in the identical dose that was chosen for the global pivotal studies. As a further layer of evidence, the final dose and schedule approved by each regulatory body in NA/EU and Asia were identical for every new MTA approved since 2001 (Table 1). While our study only focused on the impact of race and dose selection of 17 MTA that were approved over the last 15 years by all three major regulatory agencies, the drug disparity gap widens when we take into account MTA that have been approved in North America and/or Europe (e.g., cabozantinib, palbociclib, neratinib, rucaparib, vismodegib, brigatinib, and others) but remain to be approved in Asia.

To further underscore these findings, we compared the results of two phase I trials of DS-7423, a PI3K/mTOR dual inhibitor, conducted in parallel settings in predominantly Caucasian (US) and Asian (Japan) patients. Consistent with our retrospective findings, an important outcome of these phase I studies is the equivalent findings for PK, PD, safety, and final RP2D between Caucasian and Asian patients. The only significant differences were in BW and race. Oncology drugs have historically been dosed according to BSA, with the assumption that BSA-based dosing reduces inter-patient variation in drug exposure and provides a better therapeutic index.^49^ Although studies of numerous cytotoxic drugs have shown that BSA-based dosing is not superior to flat dosing, the dogma of this practice persists. Here, this study in US and Japanese populations demonstrates that, despite differences in BW and BSA, flat dosing of MTA does not affect drug exposure or toxicity.

A second critical finding here is that race or ethnicity did not impact on drug metabolism, toxicity, or efficacy. In the Japanese study, all patients identified themselves as Asian, while in the US study, 90% of patients identified themselves as Caucasian. While “racial/ethnic differences” are generally understood as differences in pharmcogenomics such as polymorphisms in drug metabolic enzymes or drug transport, this is much more complex and includes variables such as diet, smoking, alcohol, concomitant medications, supplements, and compliance. Our study only evaluates molecularly targeted drugs and not applicable to cytotoxic oncology drugs. In cytotoxic drugs the example of tegafur (FT) is an apt one. FT is a prodrug of 5-fluorouracil (5-FU), and it is mainly catalyzed by the liver microsomal enzyme cytochrome P450 2A6 (CYP2A6). It is known that CYP2A6*7, *8, *10, and *11 are highly prevalent in the Japanese population.^50^ Therefore, decreased metabolism from FT into 5-FU was anticipated in Asian patients. However, the reality was that there was no difference in PK of 5-FU while metabolism of FT was influenced by CYP2A6 polymorphisms.^51^ These reports suggest that PK of 5-FU may not be mainly influenced by CYP2A6 polymorphisms,^52–54^ and other factors, such as individual creatinine clearance and BSA, should be taken into consideration. While ethnic differences can impact on safety and efficacy and are appropriately included in drug product labels, there are currently no companion diagnostic markers to make dosing decisions at the level of individual patients. To complicate this further, there is great variability in cytochrome polymorphisms within a single ethnic group. While CYP2C8, CYP3A4, and CYP3A5 are involved in the metabolism of DS-7423 and polymorphisms in these enzymes between ethnic groups are well documented, this study did not show any differences in PK, PD, or toxicities. Therefore, we do not dispute that race and pharmacogenomics play critical roles in drug metabolism, toxicity, and efficacy. However, a specific dose evaluation might be exceptionally recommended for the specific drugs in a specific ethnicity, if pre-clinical studies were to demonstrate biomarkers that were unique to certain ethnic or racial populations that could potentially predict different outcomes.

Others have shown that 32% of drugs across all therapeutic classes have a higher dose approved in NA/EU than in Asia and, in 19% the approved dose in NA/EU is double that in Asia. Such differences are particularly notable for cardiovascular and central nervous system drugs.^55^ However, for oncology drugs, the impact of race appears to be marginal and limited to a few cytotoxic drugs. Among cytotoxic drugs, only four are approved at different doses in Asia and North America/Europe, namely, capecitabine, temozolomide, thalidomide, and arsenic trioxide.^56^ The different approved doses in these drugs were caused by dose setting in clinical trials used for application for approval in each country rather than different PK, despite the differences in polymorphism of drug metabolic enzyme among ethnicities.

Although we confirmed PI3K/mTOR inhibition by observing on-target toxicities as well as PD endpoints such as 18F-FDG-PET, blood glucose, C-peptide, and Akt phosphorylation, the lack of objective responses has led to no further development of DS-7423. This lack of significant activity can likely be attributed to two factors. First, we did not select patients harboring mutations in the PI3K-Akt-mTOR pathway. However, other studies that molecularly selected patients have only shown modest responses, suggesting that targeting this pathway may be complex and that histology and secondary mutations may dictate sensitivity to these drugs.^57^ A second factor may be the narrow therapeutic index of dual PI3K/mTOR inhibitors. By attempting to target both PI3K and mTOR, there are increased off-target toxicities, thus achieving only sub-therapeutic inhibition of PI3K and mTOR. There is now an increasing understanding that isoform-specific PI3K inhibitors may offer a more attractive anti-cancer strategy.

Nevertheless, our findings raise an important question about how we should conduct phase Ib to III and seamless trials, while recognising that race does not impact on dose selection for MTA. A global clinical trial design from early-phase to late-phase drug development and post-marketing pharmacovigilance are desperately in need. As we increasingly subtype cancer by molecular signatures, adequately powered clinical trials are only feasible with global cooperation. Signs of innovation are already apparent in China with the issuance of draft guidelines and policies that help streamline and accelerate precision medicine.^58^ Global cooperation between multiple players, such as regulatory agencies, industry, and academics, is critical to make drug development sustainable and provide timely access to life-saving drugs to cancer patients.

## Electronic supplementary material

supple fig 1A

supple fig 1B

supple fig 2A

supple fig 2B

supple fig 3

supple fig 4A

supple fig 4B

Supple Table1 eligibility

Supple Table 2 PK of MTAs

Supple Table3 DLTdetermination

APC form

## Availability of data and material

Data generated or analysed during this study are included in this published article and its supplementary information files. Further raw data are available from the authors upon reasonable request and with permission of Daiichi Sankyo Co., Ltd.
