# Supplementary figures and images for: Impact of race on dose selection of molecular-targeted agents in early-phase oncology trials
Source: Br J Cancer. 2018 May 24;118(12):1571–9. doi: 10.1038/s41416-018-0102-1 (PMC6008299; doi:10.1038/s41416-018-0102-1)

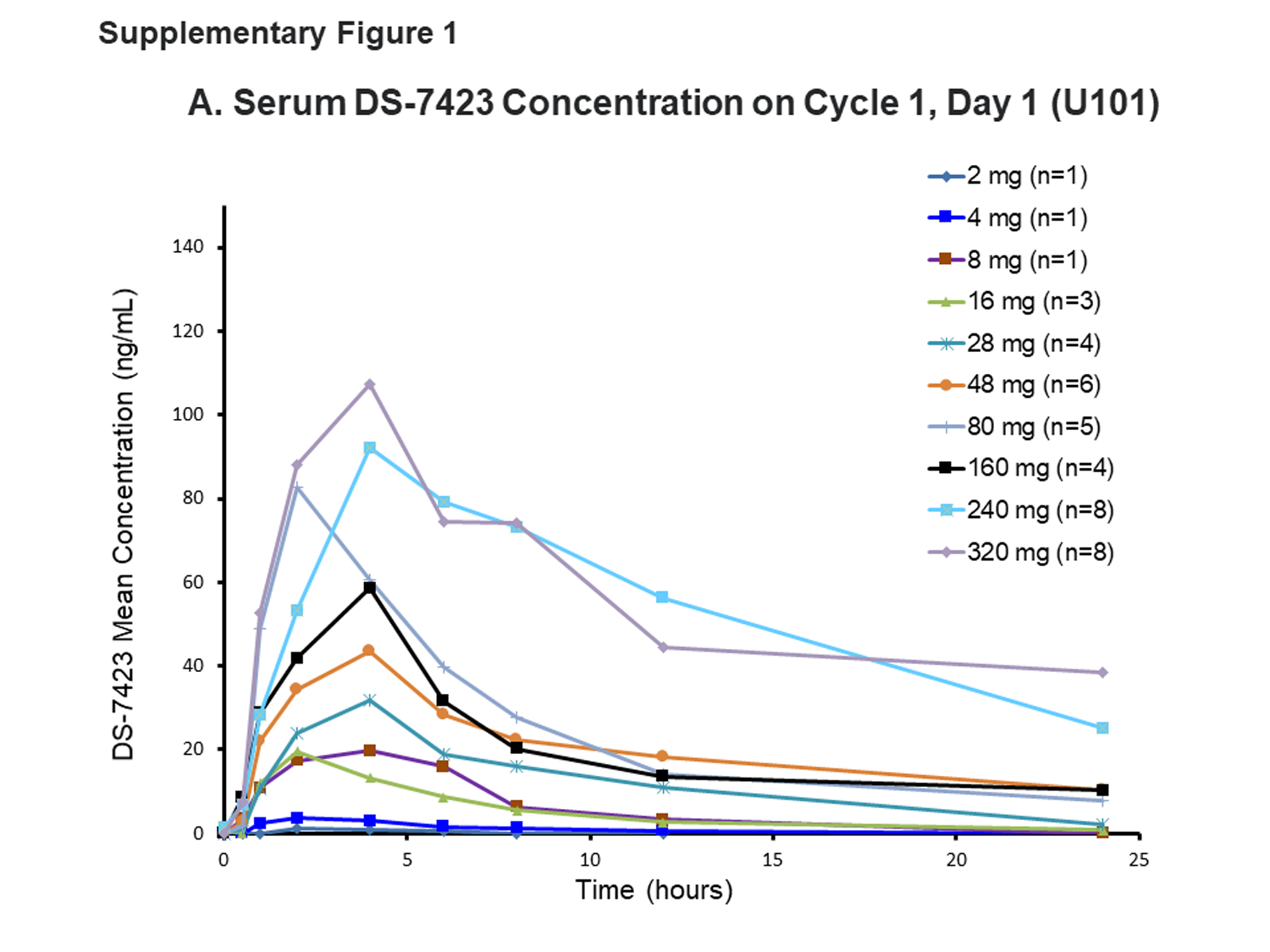

Supplement: Supplementary file 1 — supple fig 1A [file 41416_2018_102_MOESM1_ESM.tif]

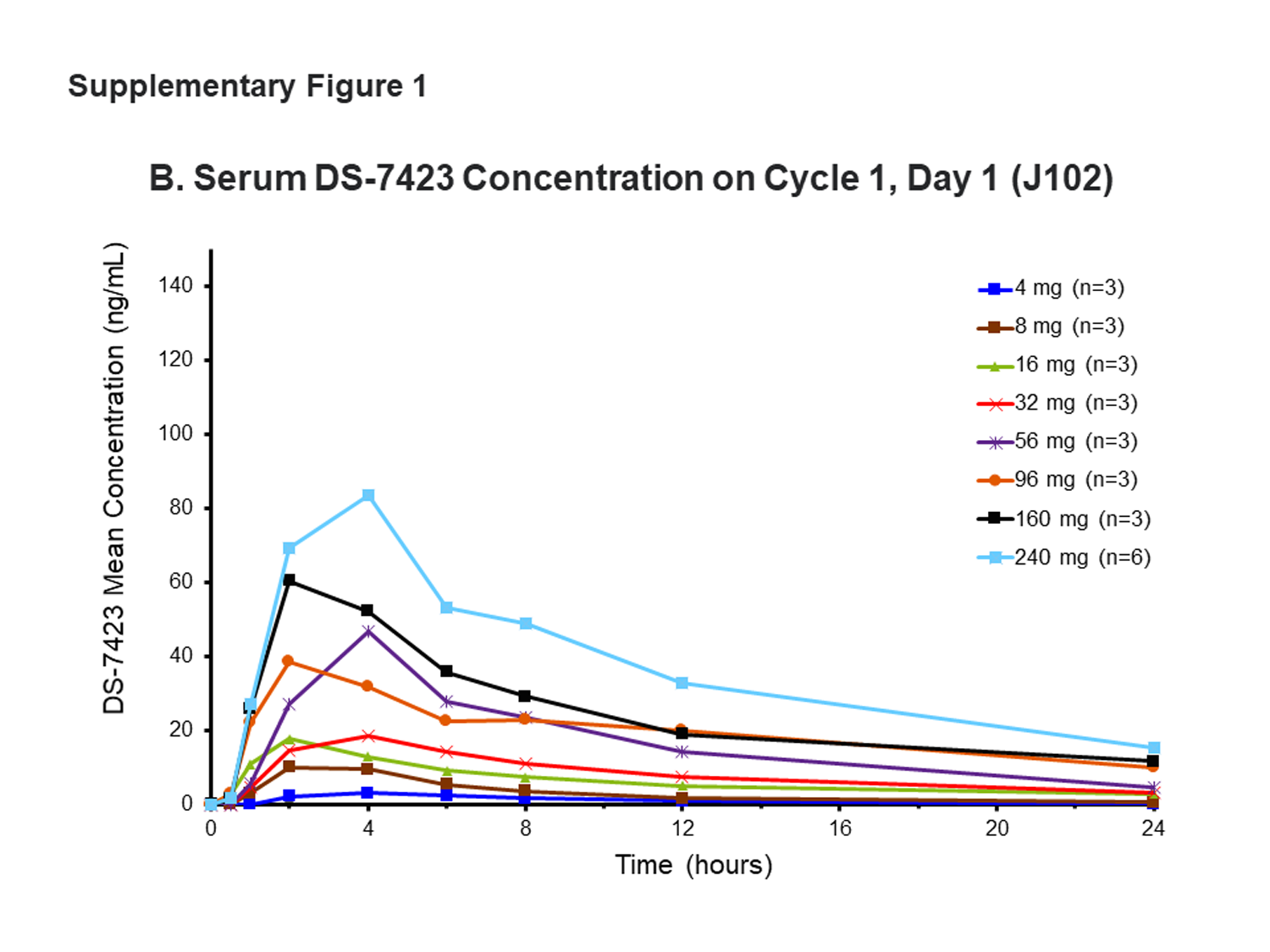

Supplement: Supplementary file 2 — supple fig 1B [file 41416_2018_102_MOESM2_ESM.tif]

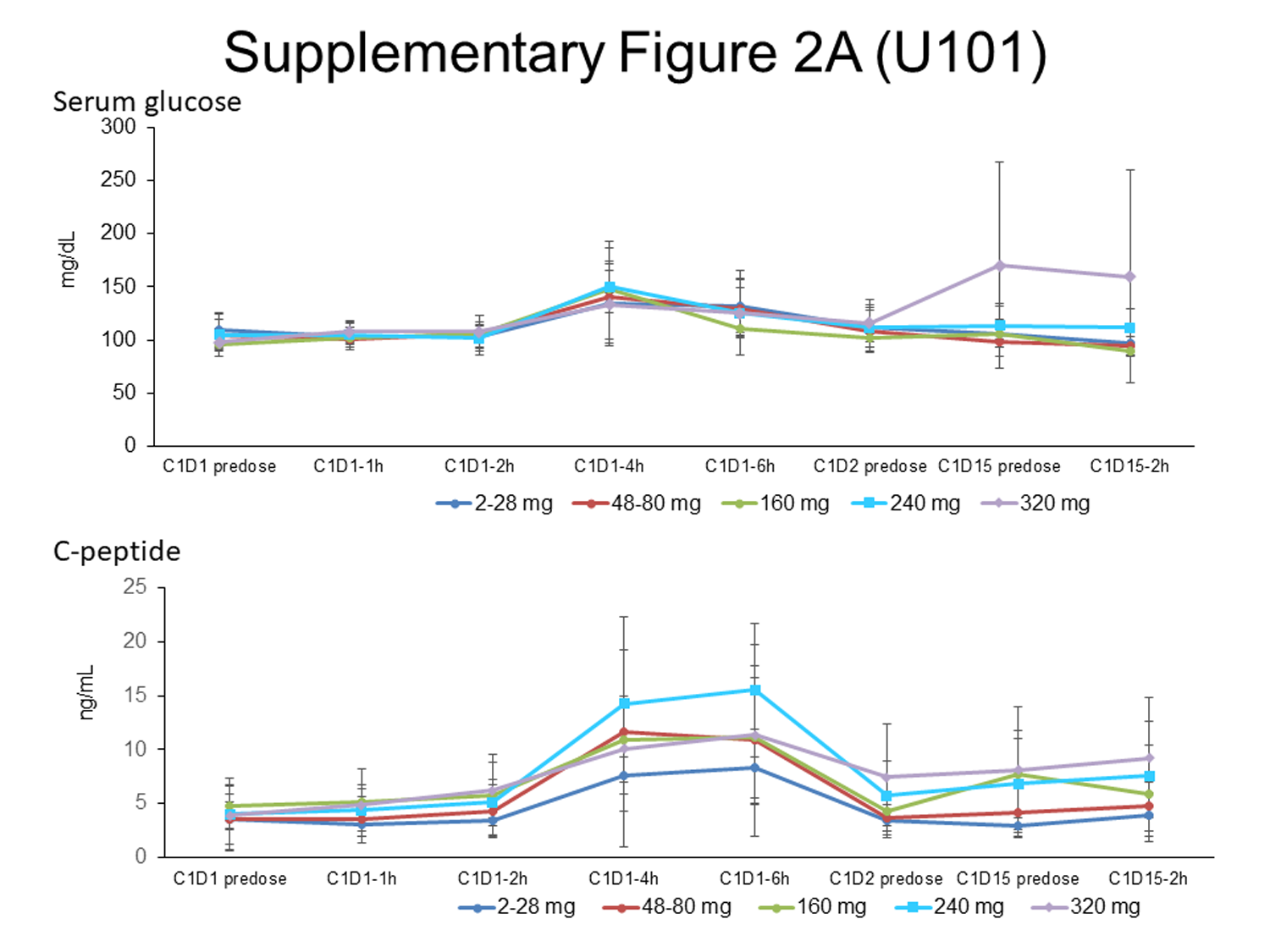

Supplement: Supplementary file 3 — supple fig 2A [file 41416_2018_102_MOESM3_ESM.tif]

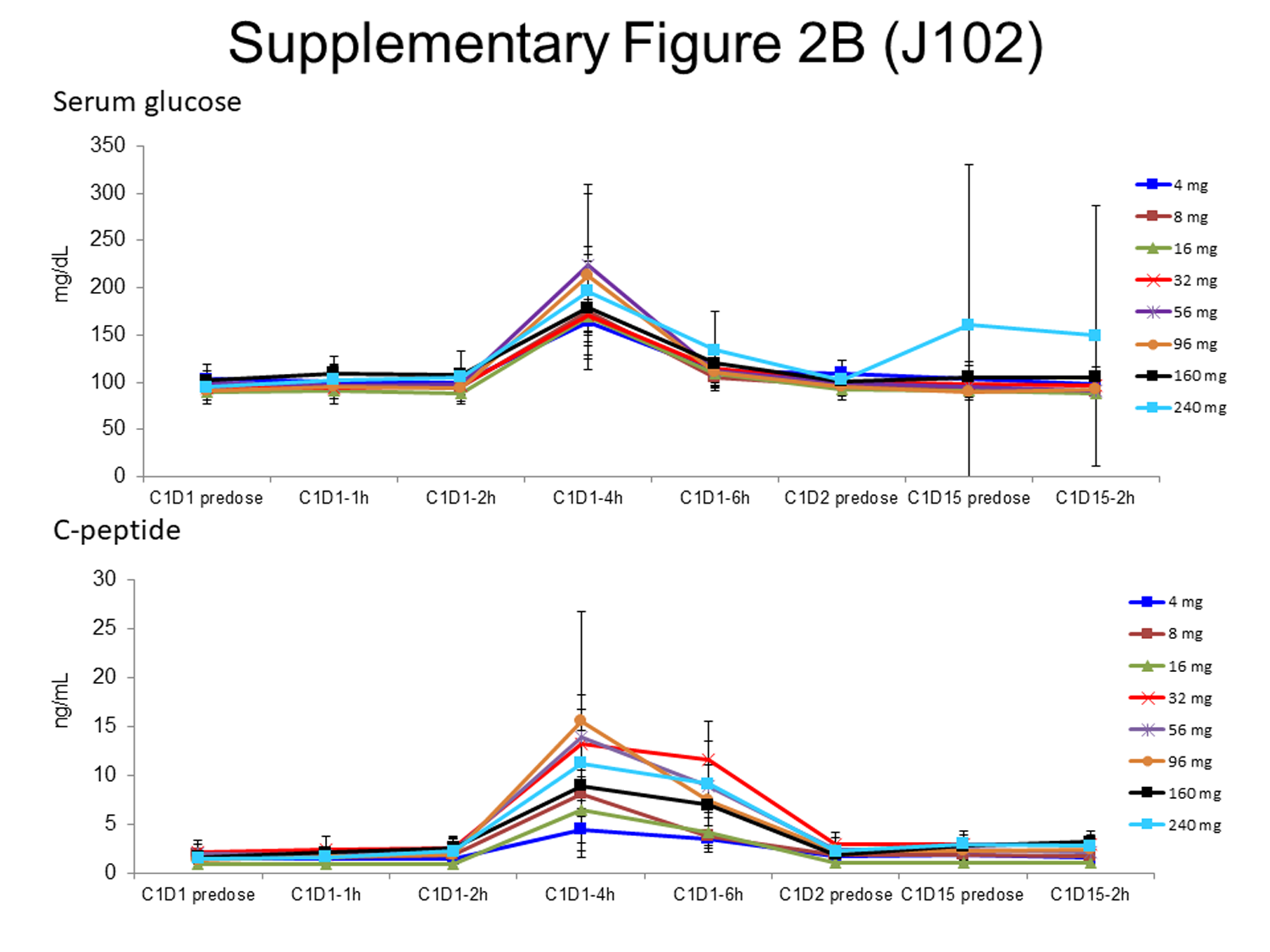

Supplement: Supplementary file 4 — supple fig 2B [file 41416_2018_102_MOESM4_ESM.tif]

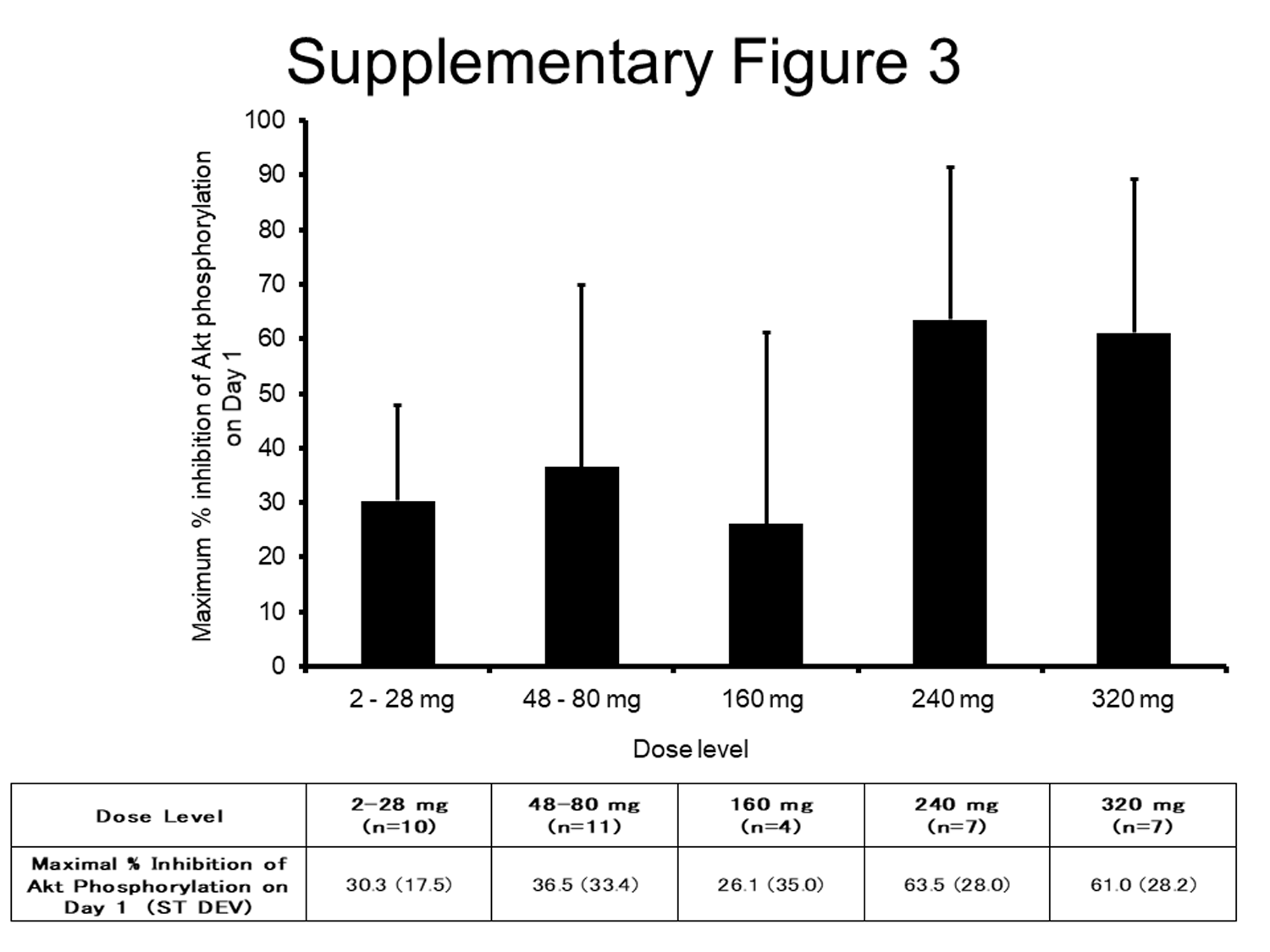

Supplement: Supplementary file 5 — supple fig 3 [file 41416_2018_102_MOESM5_ESM.tif]

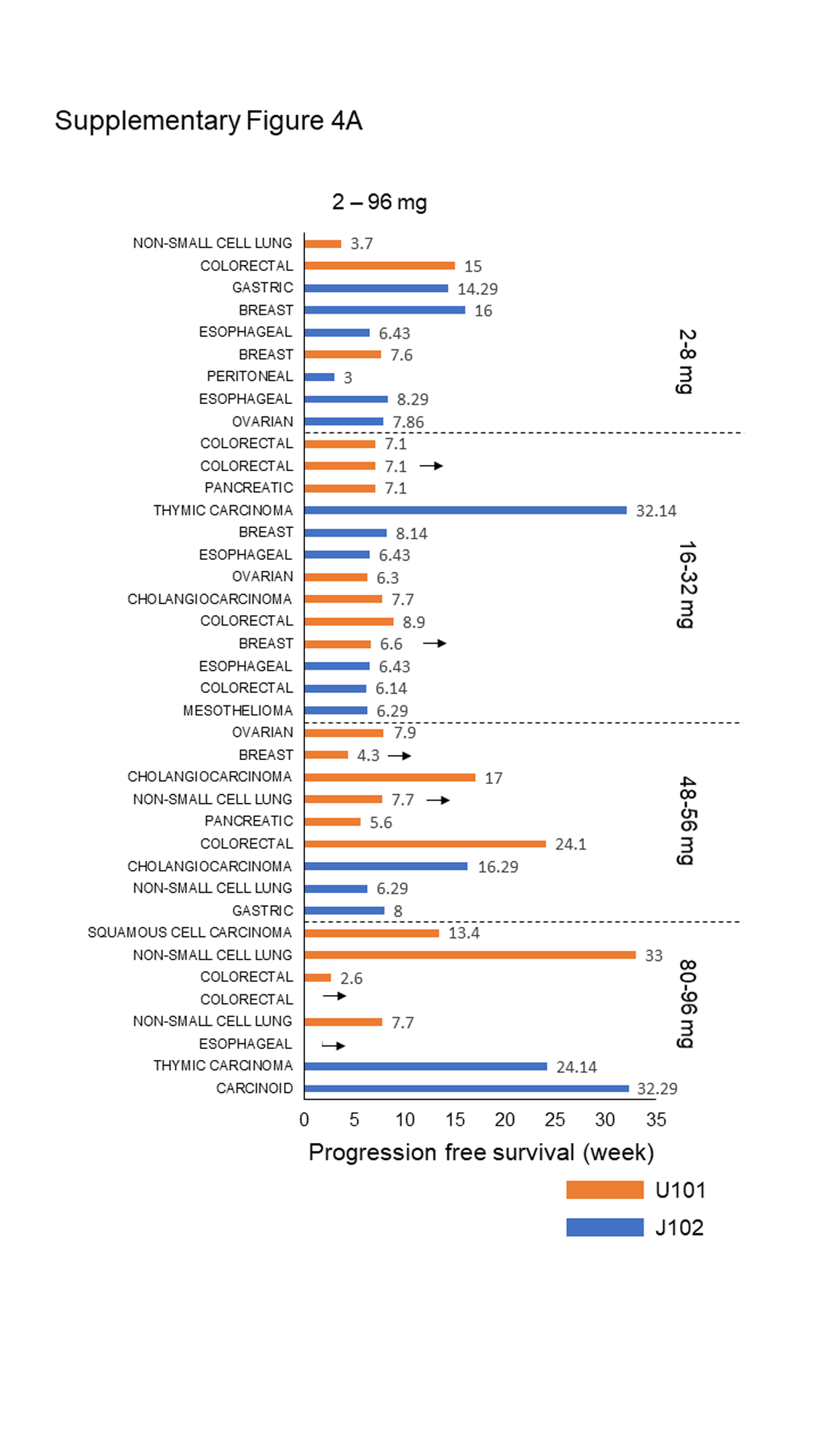

Supplement: Supplementary file 6 — supple fig 4A [file 41416_2018_102_MOESM6_ESM.tif]

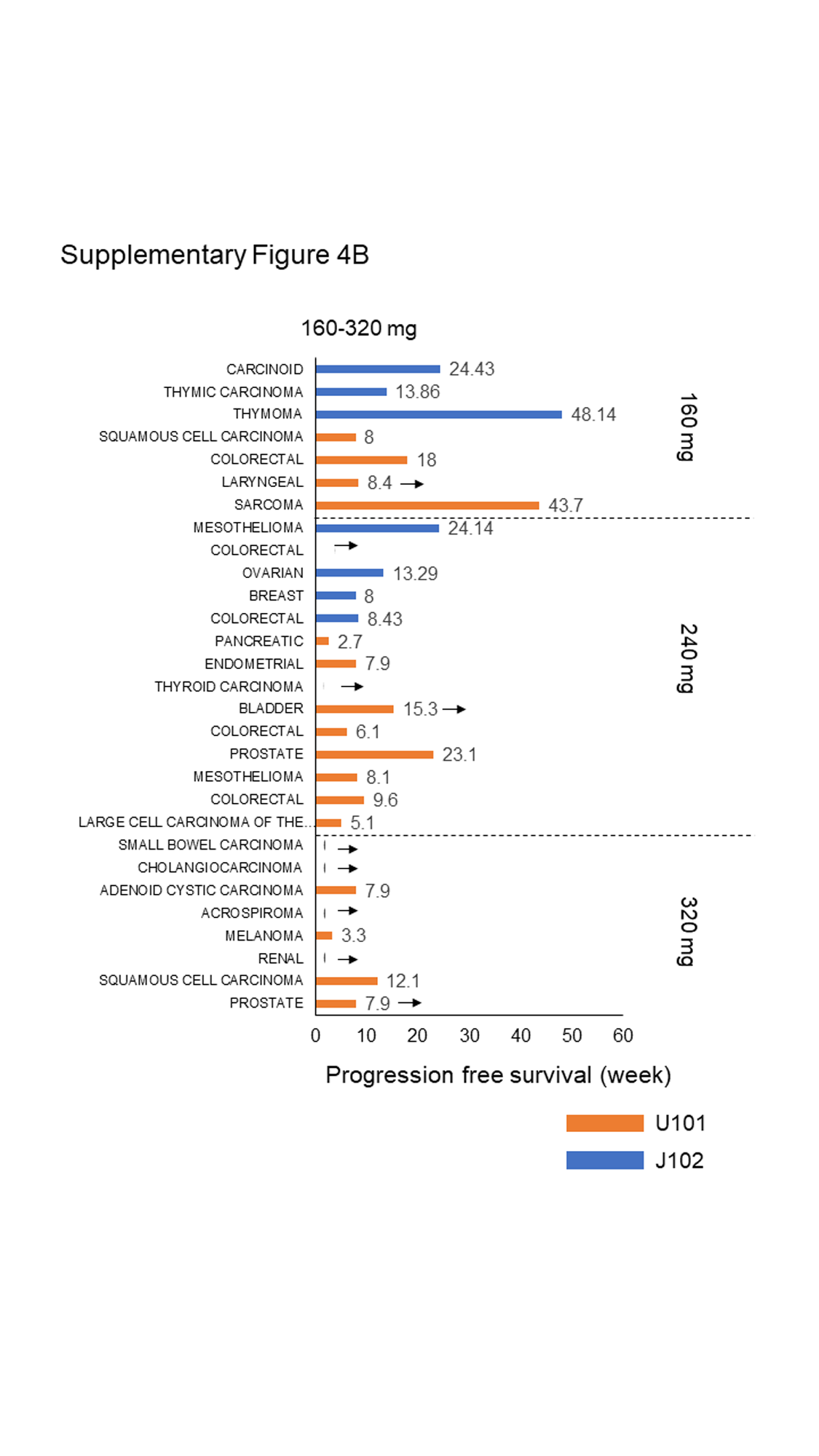

Supplement: Supplementary file 7 — supple fig 4B [file 41416_2018_102_MOESM7_ESM.tif]
