# Supplementary material for: Impact of race on dose selection of molecular-targeted agents in early-phase oncology trials
Source: Br J Cancer. 2018 May 24;118(12):1571–9. doi: 10.1038/s41416-018-0102-1 (PMC6008299; doi:10.1038/s41416-018-0102-1)
Supplement: Supplementary file 8 — Supple Table1 eligibility [file 41416_2018_102_MOESM8_ESM.docx]

# Supplementary Table 1.

| Japan study (J102) | | US study (U101) |  |
| --- | --- | --- | --- |
| **EXCLUSION CRITERIA** | | | |
| Avoid intercourse during the study and for 6 months after the last dose of the study drug | | Avoid intercourse during the study and for 90 days after the last dose of the study drug | |
| Oral intake available | | NA | |
| At least 3-month expected survival | | NA | |
| NA | | Be willing to provide available preexisting diagnostic or resected tumor samples | |
| HbA1c > 6.5% (JDS value) | | HbA1c > 7.0% | |
| Anticancer therapy within 4 weeks | | Anticancer therapy within 3 weeks | |
| Other study drug tested within 4 weeks | | Participation in a clinical study within 3 weeks | |
| NA | | History of second malignancy and primary CNS malignancies | |
| NA | | Subjects requiring daily supplemental oxygen | |
| NA | | Substance abuse or medical, psychological, or social conditions that may interfere with study participation | |
| Has complication and/or medical history within 6 months as follows: heart failure, myocardial infarction, cerebral infarction, unstable angina, arrhythmia requiring therapy, bypass surgery of artery, cerebral thrombosis, PTE, DVT, or other clinical significant cardio/pulmonary disorders | | NA | |
| Infection requiring systemic therapy, chronic diarrhea, inflammatory bowel disease, partial intestinal obstruction, retention of fluid requiring therapy, uncontrollable hypertension, or psychological symptom | | NA | |
| HIV-positive | | NA | |
| Experienced blood transfusion within 4 weeks | | NA | |
| Wide digestive resection | | NA | |

JDS: Japan Diabetes Society; NA: not applicable; CNS: Central nervous System; PTE: pulmonary thromboembolism; DVT: deep vein thrombosis; HIV: Human immunodeficiency virus
