# Supplementary material for: Impact of race on dose selection of molecular-targeted agents in early-phase oncology trials
Source: Br J Cancer. 2018 May 24;118(12):1571–9. doi: 10.1038/s41416-018-0102-1 (PMC6008299; doi:10.1038/s41416-018-0102-1)
Supplement: Supplementary file 9 — Supple Table 2 PK of MTAs [file 41416_2018_102_MOESM9_ESM.docx]

# **Supplementary Table 2. Comparison of the** **pharmacokinetic parameters of approved drugs between Caucasian and Asian**

| Name of Drug | MTD | AUC_0-24_ [ng·h/mL] | | C_max_ [ng/mL] | | T_1/2_ [h] | | T_max_ [h] | | Day |
| --- | --- | --- | --- | --- | --- | --- | --- | --- | --- | --- |
|  |  | Caucasian | Asian | Caucasian | Asian | Caucasian | Asian | Caucasian | Asian |  |
| Afatinib | 50 mg QD | 762* | 539 | 27.0 | 44.4 | 21.9 | 14.8 | 5.08 | 3.0 | Day 1 |
|  |  | 598 | 1010 | 36.6 | 66.8 | 22.3 | 33.5 | 5.00 | 3.0 | Day 28 |
| Erlotinib | 150 mg QD | 16510±11020 | 12845.48±3774.53 | 1136±865 | 958±457.19 | 20+/-9.74 | 25.92±9.31 | 4±3.46 | 6.00±8.92 | Day 1 |
|  |  | 38420±29550 | 42678.50±20434.75 | 2120±1520 | 2384.33±932.43 | 18.18±5.74 | 27.19±9.07 | 2±0 | 1.83±0.41 | Day 23 or 24 |
| Sunitinib | 50 mg QD | 420 | 374 | 27.7 | 22.8 | - | - | 5.0 | 7 | Day 1 |
|  |  | 1296 | 1406 | 72.2 | 69.3 | - | - | 8.5 | 6 | Day 28 |
| Axitinib | 5 mg BID | 188 | 142 | 22 | 17.0 | 2.3 | 4.8 | 3.2 | 4.10 | Day 1 |
|  |  | 258 | 138 | 27 | 21.4 | 2.3 | 4.04 | 2.4 | 1.37 | Day 15 |
| Regorafenib | 160 mg QD | 58270 | 33042.8 | 3904 | 2522.2 | 22.23 | 30.4 | 5.033 | 3.6 | Day 21 |
| Ceritinib | 750 mg QD | 16,500±4750 | 25700 | 800±205 | 1220 | 41 | - | 4-6 | 6.96 | Day 8 |
| Lenvatinib | 24 mg QD** | 3913.8 | 3150 | 598.0 | 418 | 5.3 | - | 1.5 | 2.0 | Day 1 |
|  |  | 4783.0 | 4140 | 579.1 | 518 | 5.8 | - | 2.8 | 2.0 | Day 28 (Caucasian), Day15 (Asian) |
| Vemurafenib | 960 mg BID | 27000*** | 32900*** | 4800 | 6400 | - | 12.7 | 5.0 | 3.88 | Day 1 |
|  |  | 392200*** | 497000*** | 61400 | 73300 | 34.1 | 60.8 | - | 2.12 | Day 15 |

AUC: area under the curve

*: AUC0-∞; **:25 mg QD in Caucasian; ***: AUC0–8 h
