# Supplementary material for: Impact of race on dose selection of molecular-targeted agents in early-phase oncology trials
Source: Br J Cancer. 2018 May 24;118(12):1571–9. doi: 10.1038/s41416-018-0102-1 (PMC6008299; doi:10.1038/s41416-018-0102-1)
Supplement: Supplementary file 10 — Supple Table3 DLTdetermination [file 41416_2018_102_MOESM10_ESM.docx]

**Supplementary Table 3. Number of patients (n) at each dose level and corresponding Dose Limiting Toxicities**

| Dose level (mg) | U101, n | U101 patients with DLT, n | J102, n | J102 patients with DLT, n |
| --- | --- | --- | --- | --- |
| 2 | 1 | - | - | - |
| 4 | 1 | - | 3 | - |
| 8 | 1 | - | 3 | - |
| 16 | 3 | - | 3 | - |
| 28 | 3 | - | - |  |
| 32 | - | - | 3 | - |
| 48 | 6 | G3 Macular rash, 1 | - |  |
| 56 | - | - | 3 | - |
| 80 | 3 | - | - | - |
| 96 | - | - | 3 | - |
| 160 | 3 | - | 3 | - |
| 240 | 7 | G3 Stomatitis, 1 | 6 | G4 Hyperglycemia, 1  G3 Lung infection, 1 |
| 320 | 6 | G3 Fatigue, 1  G3 Dehydration, 1 | - |  |
