# Supplementary material for: Impact of race on dose selection of molecular-targeted agents in early-phase oncology trials
Source: Br J Cancer. 2018 May 24;118(12):1571–9. doi: 10.1038/s41416-018-0102-1 (PMC6008299; doi:10.1038/s41416-018-0102-1)
Supplement: Supplementary file 11 — APC form [file 41416_2018_102_MOESM11_ESM.docx]

## Supplementary Figure Legends

## Supplementary Figure 1

Plasma pharmacokinetic profile of DS-7423 in patients with advanced solid tumours. Each line represents the mean DS-7423 plasma concentration measured on day 1, cycle 1, in U101 (A), and on day 1, cycle 1, in J102 (B). Time scale is hours after dose.

## Supplementary Figure 2

Serum glucose and C-peptide concentrations at predose, 1, 2, 4, 6, and 24 h after dosing on day 1, and predose and postdose on day 15 in U101 (A) and J102 (B). Data are shown as mean ± SD.

## Supplementary Figure 3

A plot of the maximal percent inhibition of TRAP-stimulated Akt phosphorylation in platelets on day 1 from baseline (n=39). The maximal inhibition was calculated by choosing the lowest percent of phosphor-Akt at 1, 2, 4, or 6 h relative to the baseline values taken as 100%. Patients were grouped into the five dose levels and means of the values and SD were plotted against those dose levels.

## Supplementary Figure 4

Swimmer plots of progression free survival in studies at 2–96 mg (A) and 160–320 mg (B).
